# Alternative Nasal Reconstruction Technique Using a Paramedian Forehead Flap and Intranasal Full-Thickness Skin Graft in a Patient With Recurrent Basal Cell Carcinoma and Previous Contralateral Forehead Flap: A Case Report

**DOI:** 10.7759/cureus.100432

**Published:** 2025-12-30

**Authors:** Alhan Fernando Castillo Valencia, Michael Johannes Hirsch Meillon, Oswaldo Paul Sánchez Alvarez

**Affiliations:** 1 Surgery, Hospital Regional Universitario de Colima, Colima, MEX; 2 Plastic and Reconstructive Surgery, Instituto Estatal de Cancerología de Colima, Colima, MEX

**Keywords:** basal cell carcinoma, frontal flap, nasal reconstruction, paramedian flap, reconstructive surgery

## Abstract

The nose is one of the facial regions most commonly affected by skin cancer, and its reconstruction represents a dual aesthetic and functional challenge. When defects involve more than 50% of a subunit or multiple aesthetic units, reconstruction requires thin, flexible tissue with reliable vascularity. The paramedian forehead flap remains one of the most versatile options, particularly in recurrent tumors, where achieving clear margins and restoring nasal patency is essential.

A 54-year-old male with a history of solid infiltrative basal cell carcinoma of the right nasal ala, previous paramedian forehead flap reconstruction, and positive margins underwent evaluation. Comorbidities included HIV with an undetectable viral load, diabetes mellitus type 2, hypertension, alcoholism, and smoking. Examination revealed a 10 × 15 mm, pearly, ulcerated lesion with telangiectasias. After tumor excision, a paramedian forehead flap was designed and elevated with a 2-cm vertical pedicle. A retroauricular full-thickness skin graft was fashioned into a tubular structure and sutured to the healthy nasal vestibule to re-create the right neovestibule. The flap was inset through an opening in the forehead flap, and a cotton-filled glove finger was placed intranasally for support. The patient progressed favorably with adequate flap vascularization.

Paramedian forehead flaps remain the gold standard for large nasal defects due to their axial blood supply and color match. The addition of tubular full-thickness grafts expands their applicability in complex vestibular reconstruction, with literature supporting their reliability and functional outcomes.

This case highlights a versatile technique combining a paramedian forehead flap with a tubular full-thickness graft to restore nasal contour and vestibular patency. It represents a reproducible strategy for complex nasal reconstruction with promising early aesthetic and functional results.

## Introduction

The nose is one of the facial units most frequently affected by skin cancer. Owing to its aesthetic and functional significance, it is important to propose therapeutic strategies that achieve both an optimal cosmetic outcome and a low recurrence rate [1]. Reconstructive facial surgery following a recurrence is often complex. When nasal defects involve two or more aesthetic subunits or more than 50% of a single subunit, reconstruction requires a flap that must be flexible, thin, and provide an adequate color match. The paramedian forehead flap can be a valuable option. In cases involving extensive defects, it is essential to ensure adequate functional coverage. Even microvascular flaps are of limited usefulness for this type of reconstruction [2]. 

The forehead flap has a pedicle with a strong vascular supply. Various vascular studies have confirmed the safety and clinical benefits of using the forehead flap, highlighting its anatomical adaptability as a reliable option for nasal reconstruction [3].

However, revision nasal reconstruction after a prior forehead flap presents additional technical challenges. Altered vascularity, dense scarring, and distortion of normal anatomical planes can complicate dissection, while previously used donor sites may restrict available options for secondary reconstruction. These factors demand meticulous surgical planning to achieve adequate structural support and acceptable aesthetic results [3].

There is a positive impact on the quality of life of patients undergoing facial reconstructive surgery, with improvements observed in body image perception, self-esteem, and overall well-being [4]. Performing nasal reconstruction techniques opens a perspective in which a thorough understanding of anatomy is essential, as modifications to the technique itself can serve as therapeutic strategies that lead to improved functional and aesthetic outcomes [4].

## Case presentation

We present the case of a 54-year-old male who attended the clinic with a diagnosis of infiltrative solid-type basal cell carcinoma of the right nasal ala, confirmed as malignant. His medical history was notable for tobacco and alcohol use, type 2 diabetes mellitus, systemic arterial hypertension, and HIV infection with undetectable viral load and CD4 count greater than 200.

He also had a history of basal cell carcinoma of the right nasal ala, which had been treated six months earlier with surgical resection and reconstruction using a paramedian forehead flap. Histopathological analysis of that specimen demonstrated positive surgical margins.

On physical examination, a solitary lesion was identified on the right nasal ala, measuring approximately 10x15 mm. The lesion exhibited pearly, raised borders with ulceration covered by a thin hemorrhagic crust. Prominent arborizing telangiectasias were observed along the peripheral edges. The surface was irregular but well-circumscribed, with no evidence of secondary infection.

**Figure 1:**
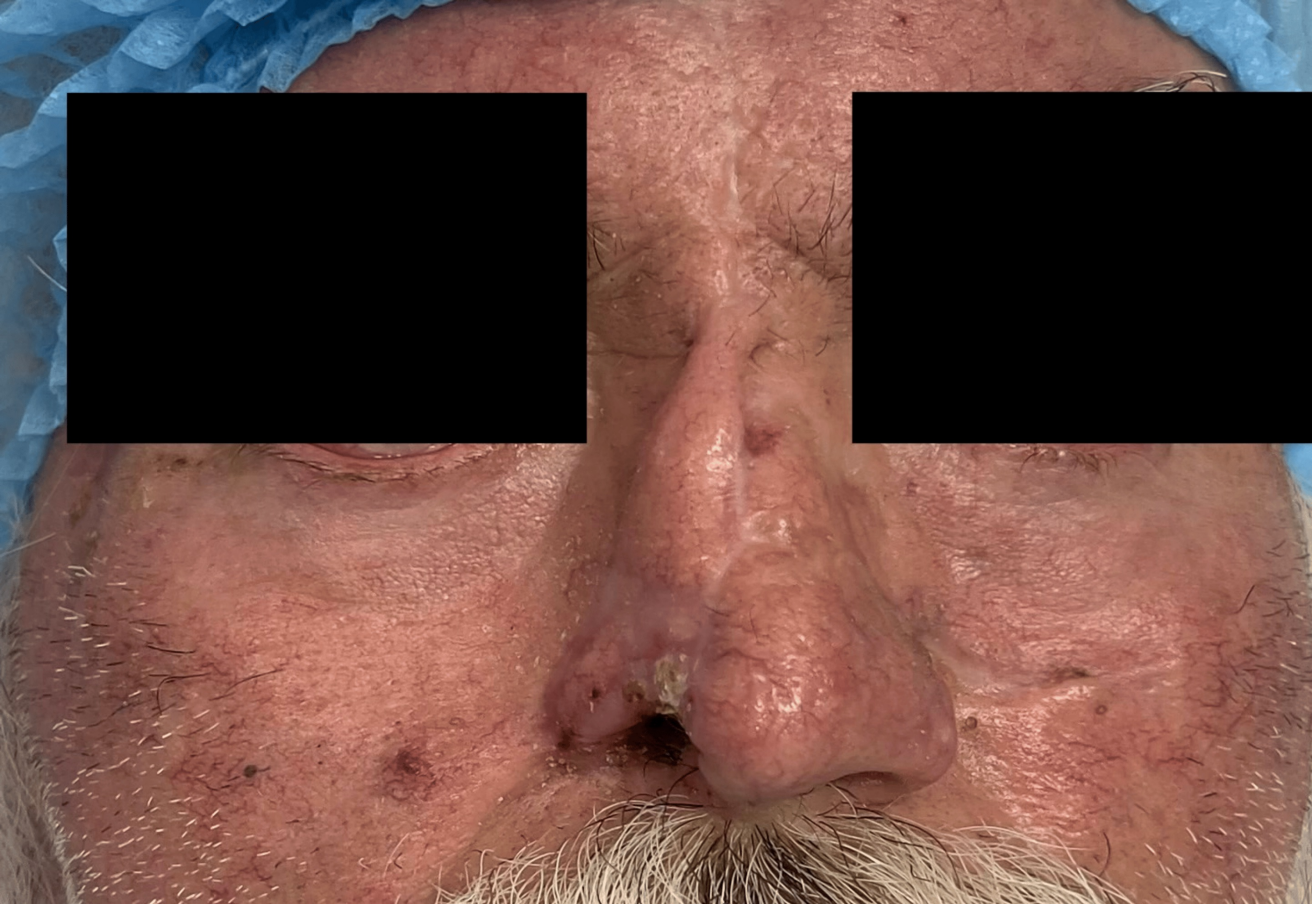
Preoperative view showing a patient with a nodular lesion on the right nasal ala and evidence of a previous contralateral forehead flap

The lesion was non-tender on palpation and presented with firm consistency. Clinical morphology was highly suggestive of nodular-ulcerative basal cell carcinoma (Figure 1).

The paramedian forehead flap was meticulously designed to match the dimensions of the nasal defect. The flap, featuring a vertically oriented pedicle measuring approximately 2 cm in width, was elevated from distal to proximal. The contralateral side was not used because the supratrochlear artery had been sacrificed during the prior procedure, rendering that pedicle unavailable for a second reconstruction. Careful dissection was performed through the subcutaneous tissue and periosteum.

**Figure 2:**
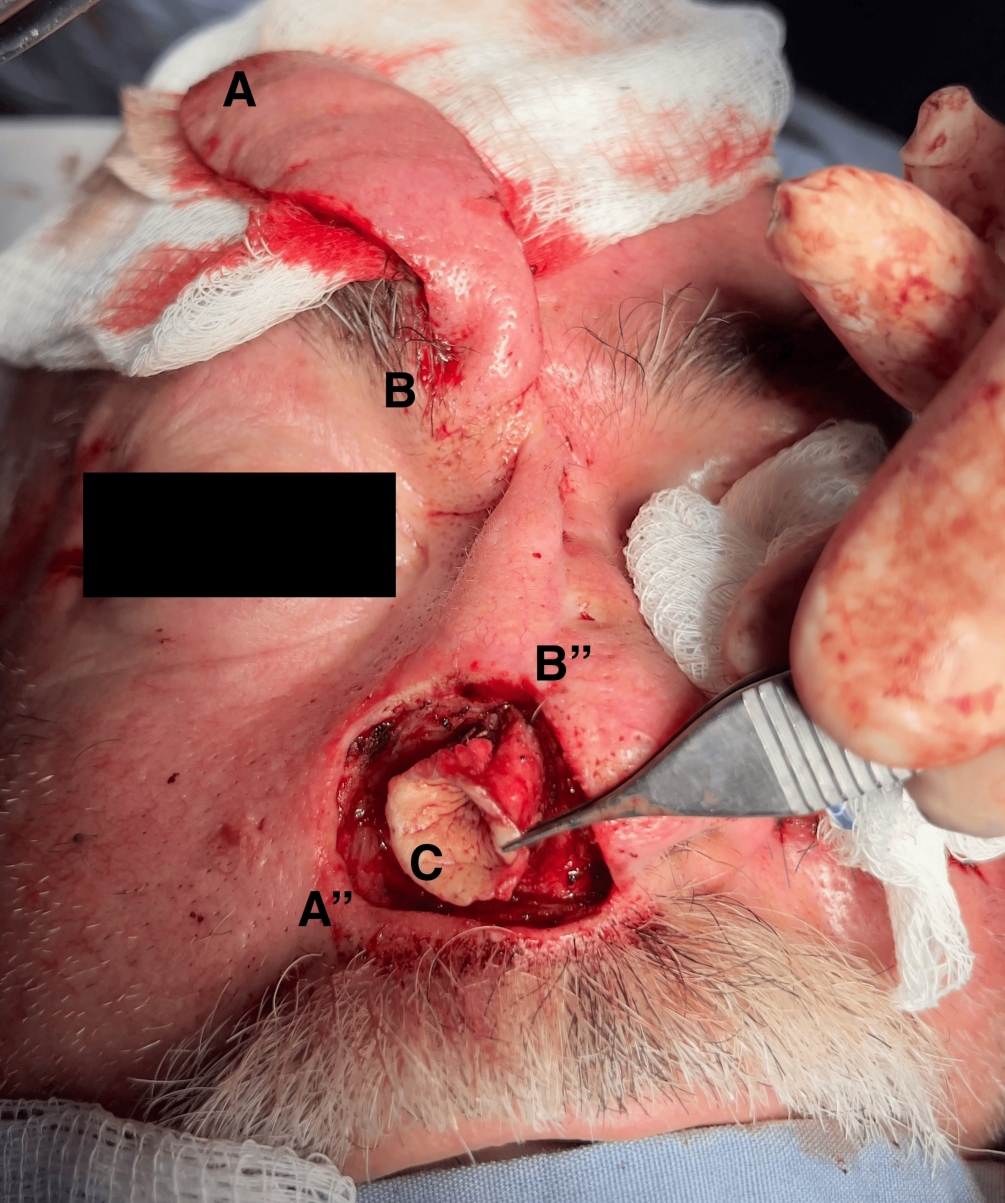
Paramedian forehead flap procedure Panels A, A′′, B, and B′′ illustrate the counterclockwise rotation of the flap, while panel C shows the tubular structure with a full-thickness skin graft sutured to the nasal antrum.

A full-thickness skin graft was harvested from the retroauricular region and fashioned into a tubular structure. The tubular structure was sutured with monofilament, simple interrupted stitches to the healthy mucosa at the base of the nasal vestibule, and cartilage harvest was not required to complement the coverage of the antral defect (Figure 2).

**Figure 3:**
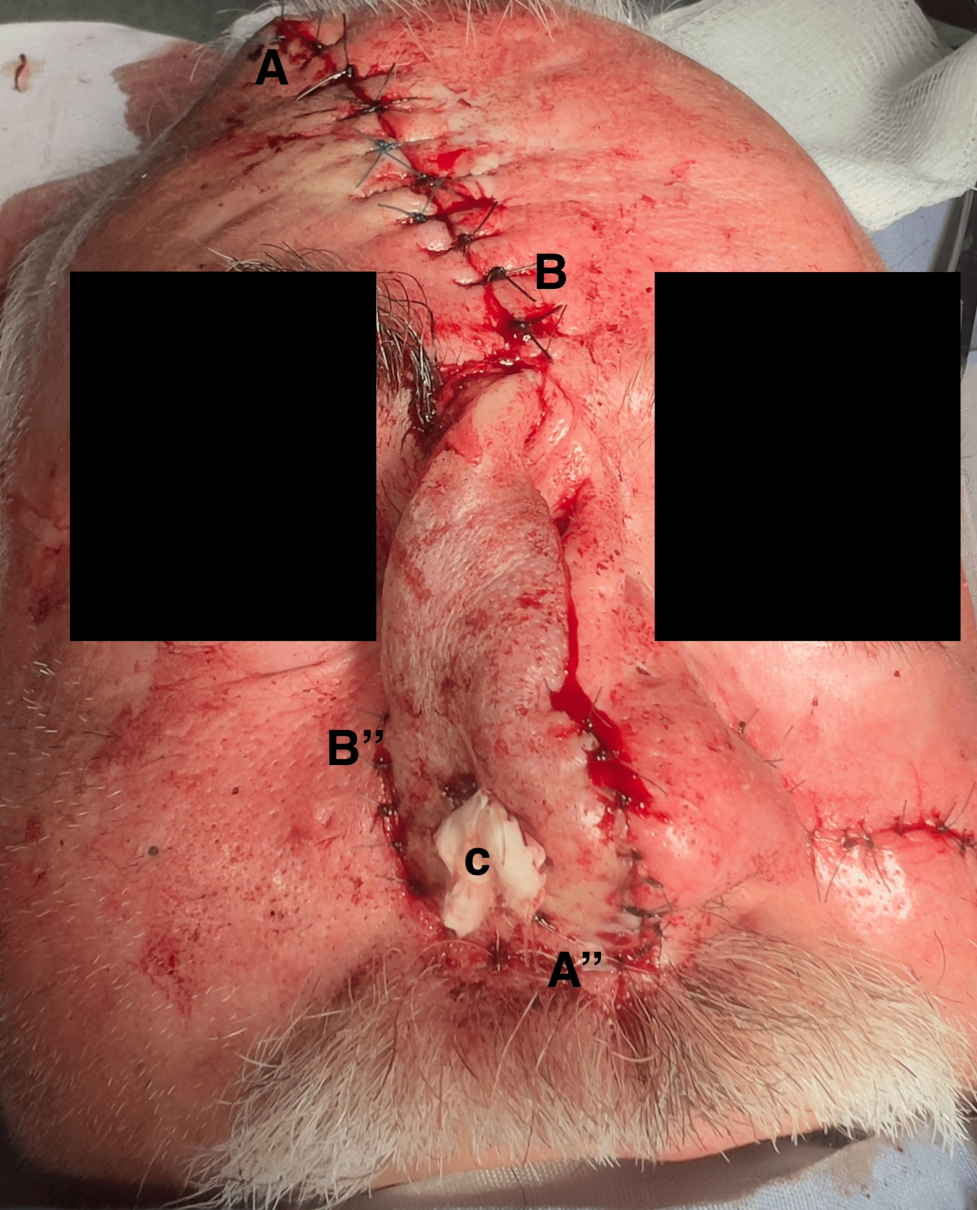
The paramedian forehead flap procedure with counterclockwise rotation of the flap (A, A′′, B, B′′). Panel C shows the opening through which the tubular full-thickness skin graft structure is exposed and sutured to the nasal antrum, with a cotton-filled glove placed inside for support.

An opening was created in the frontonasal flap to allow the tubular structure to cover the newly formed right nostril. Cotton was placed inside a sterile glove finger, and it was inserted into the newly formed nostril (Figure 3).

The patient was successfully treated with resection of the right nasal ala and vestibule, followed by coverage using a paramedian forehead flap. This case highlights the creation of a tubular full-thickness retroauricular skin graft serving as a substitute for the mucosal lining of the right nostril.

**Figure 4:**
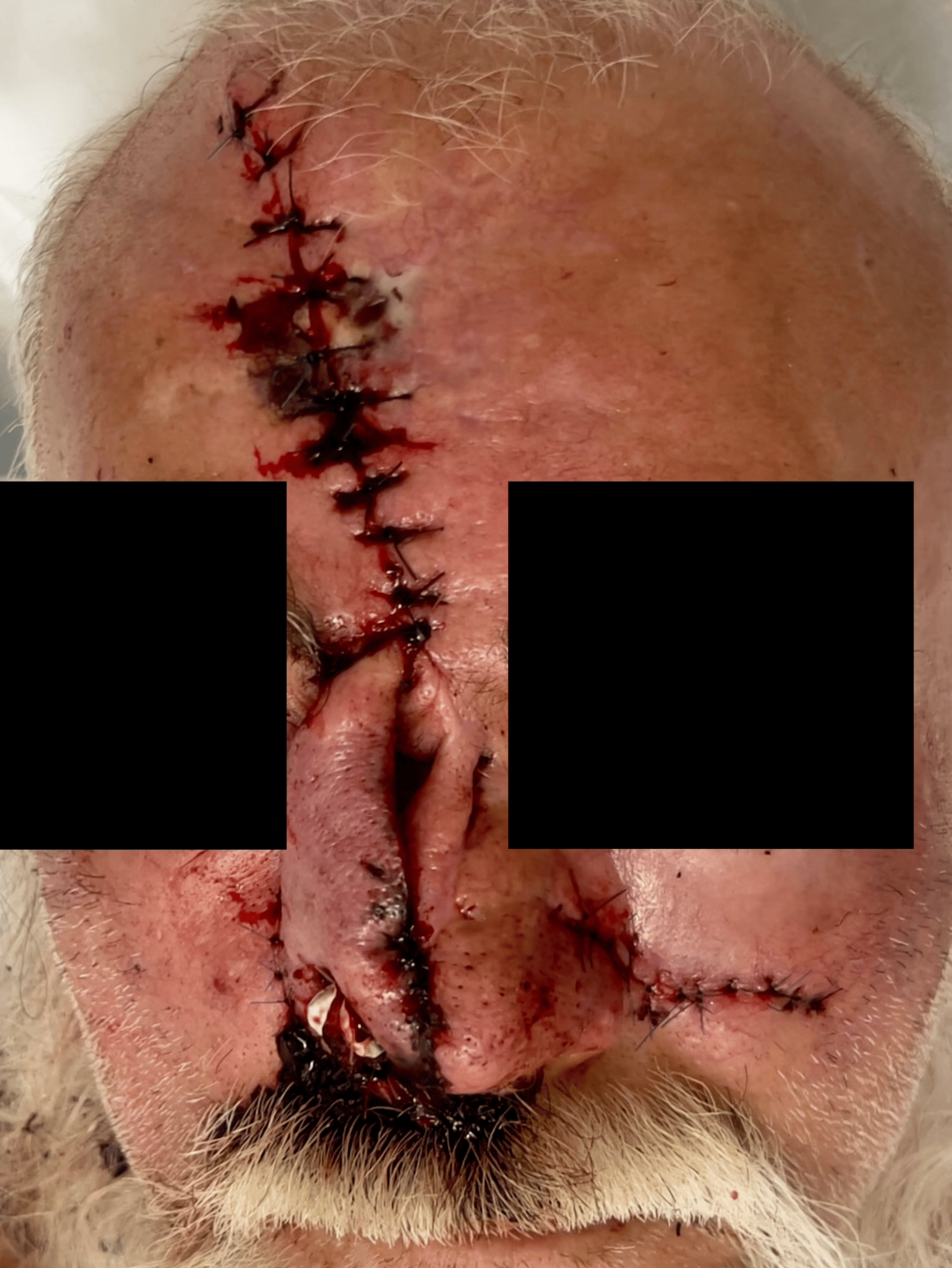
Postoperative image seven days after surgery showing a viable paramedian forehead flap

Following the flap elevation, the patient was hospitalized and underwent daily wound care for seven days, during which the flap demonstrated adequate vascularization (Figure 4). Airway patency was assessed through anterior rhinoscopy to confirm the patency of the nasal cavity and by using the Nasal Obstruction Symptom Evaluation (NOSE) scale, which yielded a score of 45 points, indicating moderate obstruction, as expected due to postoperative edema. He was subsequently discharged home.

## Discussion

The frontal flap has been used and described since ancient times. The Indian surgeon Sushruta was the first to perform a nasal reconstruction using a midline forehead flap, which, since 1600 B.C., has been known as the “Indian flap” [5,6].

Facial reconstructive surgery, specifically of the nose, represents both an aesthetic and a functional challenge due to its complex structure [5,7]. Nasal reconstruction following complete tumor excision requires a thorough understanding of the regional anatomy, as well as advanced surgical skills and experience. Depending on the involved subunits and the size of the defect, different surgical strategies may be employed [7].

The main reason for using a paramedian forehead flap is to repair large nasal defects, but it can also be applied for reconstructing periocular areas and the skull base [8].

Defects larger than approximately 1.5 to 2 cm in diameter are generally the most appropriate for this technique since smaller defects are often effectively treated with single-stage options such as bilobed flaps or skin grafts [8,9]. Some surgeons also undertake intricate, multi-stage and multi-layer reconstructions in which paramedian forehead flaps are folded or wrapped around support structures, such as cartilage or titanium plates, to re-create the entire nasal framework [9,10].

Nasal reconstruction with a paramedian forehead flap typically requires at least two surgical stages, usually performed three to four weeks apart. In some cases, a third stage is added to further refine the flap’s contour. Although harvesting the flap produces a vertical forehead scar, it is generally discreet and not highly noticeable. Overall, both the aesthetic and functional outcomes are usually very pleasing to patients and surgeons alike [10,11].

In our patient, we highlight the use of a full-thickness skin graft fashioned as a tubular structure. In our review of the literature, we did not identify reports describing this type of construct as a coverage strategy for the nasal vestibule following nasal ala resection. The advantages of this approach include increased vascularity and graft viability, ease in designing the structure, and sufficient flexibility to allow complete coverage of the nasal vestibule [11].

Among the planned strategies, we elected to harvest the cutaneous graft as the basis for vestibular lining, following the principles of mucosal coverage [11].

It was decided, as part of the strategy to complement the forehead flap, to use a full-thickness skin graft due to its ease of manipulation, flexibility, and elasticity for contouring a previously operated site, as well as the practicality of its harvest. This approach provides favorable outcomes with a shorter operative time compared to the harvest and placement of auricular cartilage.

This tubular structure was sutured to the base of the resected nasal vestibule, and cotton impregnated with sterile saline solution was placed and sutured within it so that the graft remained in full contact with the exposed mucosa.

In our experience, the frontonasal flap may be associated with different types of complications. In patients with diabetes mellitus and chronic tobacco use, as in our case, signs of arterial insufficiency may occur more frequently, including a pale, cool graft with diminished capillary refill. Venous congestion appears to a lesser extent and is characterized by edema, firmness, ecchymosis, and dark blood upon needle pricking [11].

## Conclusions

The paramedian forehead flap, combined with a full-thickness preauricular skin graft fashioned into a tubular structure, provides a reliable and versatile reconstructive option for complex nasal vestibular and external nasal defects. This technique leverages the robust axial vascularity of the supratrochlear artery while simultaneously restoring the three-dimensional architecture required for adequate nostril contour, support, and patency. Our experience demonstrates that the integration of a tubular full-thickness graft not only re-creates a functional neovestibule but also enhances airway stability during the early postoperative period, contributing to favorable healing dynamics.

Meticulous flap design, atraumatic elevation, and careful inset of the tubular graft are critical for optimizing vascular preservation and minimizing complications such as stenosis, flap congestion, or contour deformities. The early postoperative outcomes observed in this case highlight the reliability of this approach, with a well-vascularized flap, satisfactory structural support, and promising aesthetic evolution within the first week. Although long-term follow-up is essential to evaluate airway stability and final cosmetic refinement, the technique offers a reproducible, anatomically respectful, and functionally oriented solution for challenging nasal reconstruction scenarios. 

## References

[1] Cerci FB, Kubo E (2020). Nasal reconstruction after Mohs micrographic surgery: analysis of 208 cases. Surg Cosmet Dermatol.

[2] D'Antonio S, Castellaneta F, Rullo V, De Rosa A, Turco P, Grieco MP, Fabrizio T (2025). Nasal reconstruction with forehead flap: our 12 years’ experience. Plast Reconstr Surg Glob Open.

[3] Reece EM, Schaverien M, Rohrich RJ (2008). The paramedian forehead flap: a dynamic anatomical vascular study verifying safety and clinical implications. Plast Reconstr Surg.

[4] Yıldız T, Selimen D (2015). The impact of facial aesthetic and reconstructive surgeries on patients’ quality of life. Indian J Surg.

[5] Kazanjian VH (1946). The repair of nasal defects with the median forehead flap; primary closure of forehead wound. Surg Gynecol Obstet.

[6] Gandhi MA, Patil BK (2024). Sushruta: the father of surgery and ancient medical innovations. Cureus.

[7] Goldman A, Wollina U (2020). Defect closure after successful skin cancer surgery of the nose: a report of 52 cases. Acta Dermatovenerol Alp Pannonica Adriat.

[8] Zito PM, Hohman MH, Mazzoni T (2025). Paramedian forehead flaps. StatPearls [Internet].

[9] Owusu J, Nesbitt B, Boahene K (2020). Management of complicated nasal defects. Facial Plast Surg.

[10] Prohaska J, Sequeira Campos MB, Cook C (2023). Rotation flaps. StatPearls [Internet].

[11] Bednarek RS, Sequeira Campos MB, Hohman MH, Ramsey ML (2023). Transposition flaps. StatPearls [Internet].

